# A PCR-Based Approach for Early Diagnosis of Head and Neck Aspergillosis: A Pilot Study

**DOI:** 10.3390/genes15111428

**Published:** 2024-10-31

**Authors:** Thaís Ellen Chaves Gomes, Victor Coutinho Bastos, Douglas Boniek, Mário Romañach, Fernanda Faria Rocha, Roberta Rayra Martins Chaves, Ricardo Santiago Gomez

**Affiliations:** 1 Department of Oral Surgery and Pathology, School of Dentistry, Universidade Federal de Minas Gerais, Belo Horizonte 31270-901, Brazil; tec1022@gmail.com (T.E.C.G.); ferfadi@gmail.com (F.F.R.); 2 Department of Pathology, Biological Science Institute, Universidade Federal de Minas Gerais, Belo Horizonte 31270-901, Brazil; coutinhobvictor@gmail.com; 3 Department of Microbiology, Biological Sciences Institute, Universidade Federal de Minas Gerais, Belo Horizonte 31270-901, Brazil; douglasboniek@yahoo.com.br; 4 Department of Oral Pathology, School of Dentistry, Universidade Federal do Rio de Janeiro, Rio de Janeiro 21941-913, Brazil; marioromanach@yahoo.com.br; 5 Medical School, Faculdade Ciências Médicas de Minas Gerais, Belo Horizonte 30130-110, Brazil; roberta.chaves@cienciasmedicasmg.edu.br

**Keywords:** aspergillosis, *Aspergillus*, PCR, diagnosis

## Abstract

Background: Aspergillosis is a fungal disease caused by the inhalation of fungal spores of the genus *Aspergillus* spp. This fungus mainly affects the lungs but can spread and infect the maxillofacial region through the bloodstream or inoculation of the fungus after extraction or endodontic treatment, especially in the upper posterior teeth. The disease has nonspecific clinical manifestations that hinder its early diagnosis. Although the Polymerase Chain Reaction (PCR) technique holds promise as a diagnostic tool for aspergillosis, anatomopathological analysis services do not routinely adopt this method. Objectives: Therefore, the present study aimed to evaluate the applicability of PCR and standardise the techniques of preparation of biological samples for the detection of the three species: *Aspergillus niger*, *Aspergillus fumigatus* and *Aspergillus flavus.* Methods: Six samples of formalin-fixed, paraffin-embedded tissue (FFPE) with a histopathological diagnosis suggestive of aspergillosis were investigated using PCR. As a positive control for the PCR reaction, morphologically and genetically characterized cultures were used, with their sequences deposited at NCBI under accession codes MW837777 (*A. fumigatus*) and MW837779 (*A. niger*). The *A. flavus* culture used is reference RC 2053. Results: Four of the six samples evaluated were positive for *Aspergillus* spp., of which one was co-infected with *A. fumigatus* and *A. flavus* species, while two others were positive only for *A. flavus*, and one sample was positive only for *A*. *fumigatus*. Conclusions: These findings suggest that PCR can be used as an auxiliary method for diagnosing aspergillosis. However, this was a pilot study, and expansion of the sample size and the evaluation of PCR in comparison with other diagnostic tests for aspergillosis are essential to determine the accuracy of the method.

## 1. Introduction

First described in 1729 by the Italian priest and biologist Pier Antonio Micheli, the fungus *Aspergillus*, from the Latin *aspergere* (to spread), received its name because it resembles aspergillum, which is a device used in churches to sprinkle holy water [1]. *Aspergillus* is a filamentous fungus that is ubiquitous in nature and is found in soil, dust, plants, and hospital ventilation systems [2]. They are important microorganisms that produce food, medicine, and enzymes [3]. However, not all species are beneficial, and some can cause human illness. Its spores remain suspended in air for long periods and can remain viable for months in a dry environment [4,5]. The inhalation of *Aspergillus* spores can cause an opportunistic infection known as aspergillosis, which remains frequently underdiagnosed [6]. Theodor Sluyter reported the first human case of pulmonary aspergillosis in 1847 [7].

Clinical presentations of aspergillosis can range from an allergic-type disease (non-invasive) to generalised forms, leading to the risk of death (invasive form) [8]. Allergic bronchopulmonary aspergillosis and allergic fungal rhinosinusitis are non-invasive forms, whereas chronic pulmonary aspergillosis is an invasive form [8,9].

The manifestation of each clinical type depends on the virulence of the fungus and the immunological host response [10]. In immunocompetent patients, the inhalation of *Aspergillus* spores rarely causes illness, as they are eliminated from the body owing to the coordinated action between the innate and adaptive responses of the immune system [2,11]. However, the infection can spread through vascular invasion to other organs [10,12], principally in immunocompromised patients. The dissemination of *Aspergillus* may affect the nose, paranasal sinuses, and the oral cavity, which makes this infection of particular interest in dentistry [5,13]. 

Fungal sinusitis is the main manifestation of *Aspergillus* infection in the maxillofacial region in immunocompromised patients [14]. Other forms of infection include oral lesions that arise after endodontic treatment or tooth extraction, particularly in the upper posterior teeth. Clinically, lesions present as necrotic ulcers on the palate that may extend to the attached gingiva, showing a nonspecific radiographic appearance [5,15]. While sinus aspergillosis lesions can be radiographically similar to viral or bacterial rhinosinusitis or even neoplasms [16], ulcerated lesions can be confused with infections caused by other filamentous fungi, such as various *Zygomycetes* (oral mucormycosis) [17]. These overlapping clinical findings can hinder the differential diagnosis of aspergillosis and delay its early identification in the oral medicine routine [14]. 

Although *Aspergillus* spores are constantly inhaled, disease is rarely observed in immunocompetent hosts. In contrast, immunosuppressed patients (corticosteroid therapy) or critically ill patients are strongly associated with high mortality and morbidity rates caused by aspergillosis [18]. Therefore, an early diagnosis is important to provide a better prognosis through assertive therapeutic intervention [14]. Histopathological analysis and culture of microorganisms are currently the gold standards for diagnosis [8]. However, culture is a time-consuming examination, and analysis of morphological characteristics of Aspergillus culture, such as colour and type of spores, is not reliable, as these characteristics are susceptible to the influence of environmental factors, especially the culture medium and incubation temperature [19]. Histopathological analysis is only suggestive of the diagnosis because of the microscopic similarities between different fungal organisms. In addition, hyphae are not always visible on histopathological examination, and other fungal infections, such as fusariosis and mucormycosis, may have histopathological features superimposed on aspergillosis [8,20].

Therefore, it is necessary to develop a tool that can aid in the rapid and reliable diagnosis of aspergillosis. Polymerase Chain Reaction (PCR) is a potential tool for early and accurate diagnosis because its benefits have already been scientifically proven [12]. However, due to the lack of a standardised technique for *Aspergillus*, PCR is not yet widely used as a diagnostic method for aspergillosis [10,21]. In the present study, we aimed to standardise PCR assays for the detection of *A. fumigatus*, *A. flavus*, and *A. niger*, which are the main species responsible for human infections [8,10], and evaluate the reliability of this technique for the rapid diagnosis of aspergillosis.

## 2. Materials and Methods

This study was approved by the Research Ethics Committee of the Universidade Federal de Minas Gerais (CAAE: 74144623.4.0000.5149) in accordance with the principles of the 1964 Helsinki Declaration and its later amendments.

**Figure 1:**
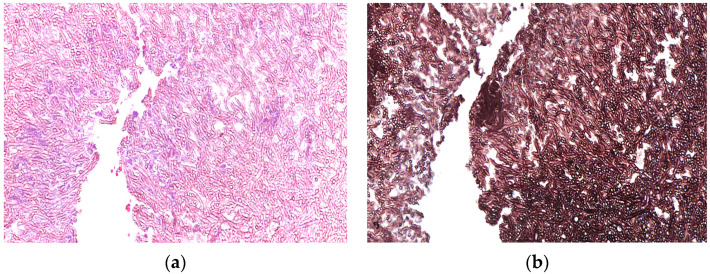
Aspergillosis histopathology. Septate hyphae, with dichotomous branching and, sometimes, forming angles of 45°, stained with H&E (**a**) and Grocott (**b**). (original magnification, H&E staining, ×40).

Digital records from the Oral and Maxillofacial Pathology Laboratory of the School of Dentistry at the Universidade Federal de Minas Gerais (UFMG) and Universidade Federal do Rio de Janeiro (UFRJ) were retrospectively assessed for all suspected aspergillosis cases between 2000 and 2023. Six samples were analysed in the present study, which were all from male patients between the ages of 52 and 79. For each case, clinical data and corresponding FFPE samples were retrieved. Cases 5 and 6 involved patients with histories of frontal and zygomatic bone fractures, respectively. The microscopic aspects of all cases were reviewed by two oral and maxillofacial pathologists (RSG and MJRGS (Figure 1)). Fungal cultures of each species were used as positive controls for the PCR. The Mycology Laboratory of the Institute of Biological Sciences at UFMG provided us with fungal cultures of *A. fumigatus* and *A. niger*, while *A. flavus* was made available by the Faculty of Veterinary Medicine at UFMG and cultivated in its own medium for seven days. An inflammatory fibrous hyperplasia sample was used as negative control.

Fungal genomic DNA (gDNA) was extracted from FFPE samples using the QIAamp DNA Blood Mini Kit (QIAGEN, Hilden, Germany) and DNeasy Blood & Tissue Kit (QIAGEN), following the manufacturer’s protocol with some modifications. The first two 5 µm thick sections were discarded, and subsequently, ten tissue sections were collected in 1.5 mL tubes and deparaffinised with 320 µL of deparaffinization solution (QIAGEN, Hilden, Germany). After adding 360 µL of ATL buffer, the samples were left at −20 °C for a minimum of 10 min. Three stainless-steel beads were added to the tubes and vortexed for 10 min to macerate the tissue and disrupt the cell walls of the filamentous fungi. After removing the beads, 40 µL of Proteinase K (QIAamp DNA Blood Mini Kit) was added, and the sample was incubated overnight at 56 °C. Then, 400 µL of AL buffer was added and vortexed for another 10 min, which was followed by incubation for 1 h at 70 °C. The entire clear phase was transferred to a new 1.5 mL tube, and 400 µL of 100% alcohol was added. The subsequent steps were performed according to the manufacturer’s protocol. gDNA extracted from purified fungal culture samples was used as a positive control following a similar protocol. Briefly, culture plates were scraped with a plastic rod and washed with sterile phosphate-buffered solution (PBS). Mycelia were collected in 15 mL tubes and centrifuged to obtain pellets. The pellet was redistributed into 1.5 mL tubes and conditioned at −20 °C until DNA extraction. Quantification and evaluation of the purity of the extracted DNA was measured on the NanoDrop^®^ 2000 spectrophotometer (Thermo Fisher Scientific, Waltham, MA, USA). The A260/280 index of the deparaffinised samples varied from 1.6 to 1.82.

PCR reactions were performed following standard procedures using MyTaq HS Red Mix, 2x (Bioline Reagents, London, UK). The optimal annealing temperature was determined by gradient PCR and was settled at 57.8 °C for all the primer sets. Cycling conditions were optimized as follows: 3 min—95 °C for initial denaturation, followed by 60 cycles (95 °C—15 s, 57.8 °C—15 s, 72 °C—30 s), and 10 min at 72 °C for final extension. Then, 300 ng of fungal gDNA was used to test the specificity of each primer set in a 25 µL PCR reaction and also served as positive control in all the experiments. For the analysis of FFPE samples, 100–200 ng of gDNA was used. The gDNA extracted from fibrous hyperplasia samples was used as negative controls in the PCR. The primer sets used in this study were designed based on a search for unique sequences of the internal transcribed spacer (ITS) regions of each species available in the NCBI database. The obtained sequences were aligned, and the most conserved regions that differentiated one genus from another were selected to guide the primer design (Table 1). Then, 1 µL of each primer (F and R, respectively, of each species) at 10 µM concentration was added to the PCR tubes. To minimise the risk of cross-contamination, reactions for each species were performed separately. PCR products were loaded onto a 1.5% agarose gel for electrophoresis, at 200 v and 200 mA for 30 min, with 5 µL of SYBR^®^ Saf DNA gel stain (Thermo Fisher Scientific, CA, USA) and subsequently inspected under UV light. PCR products were then purified using ExoSAP-IT™ PCR Product Cleanup Reagent (Applied Biosystems, Foster City, CA, USA). Bidirectional DNA sequencing was performed using a Big Dye Terminator v3.1 Cycle Sequencing Kit (Applied Biosystems) and run on an ABI3130 DNA Analyzer (Applied Biosystems). The chromatograms were manually inspected in the SnapGene Viewer software (v. 5.3.2, from GSL Biotech, San Diego, CA, USA; available at https://snapgene.com accessed on 19 September 2024). Sequences were then loaded on BLAST: Basic Local Alignment Search Tool (https://blast.ncbi.nlm.nih.gov/Blast.cgi accessed on 19 September 2024) and checked for their specificity. The GenBank accession numbers for the sequences used in the alignment were GenBank: OR939708.1 for *A. fumigatus* and GenBank: MT279323.1 for *A. flavus*.

## 3. Results

**Figure 2:**
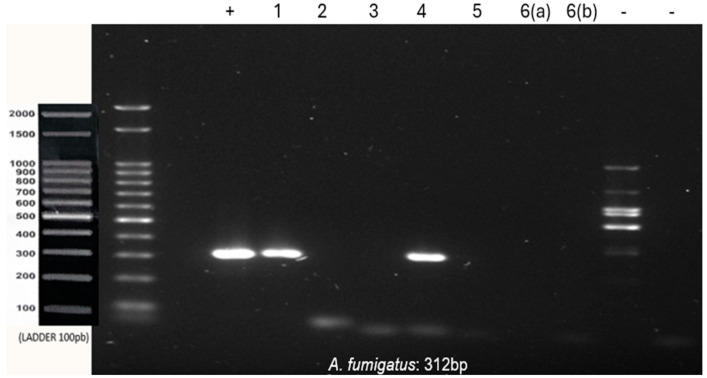
PCR using *A. fumigatus* primer with 100 ng of DNA from each sample. (+) DNA extracted from *A. fumigatus* culture (positive control). (-) Negative control of the reaction with and without human DNA. 1–6: Paraffin-embedded samples of lesions suggestive of aspergillosis. 6(a) and 6(b): Lesions removed from the same patient and embedded in two separate blocks.

**Figure 3:**
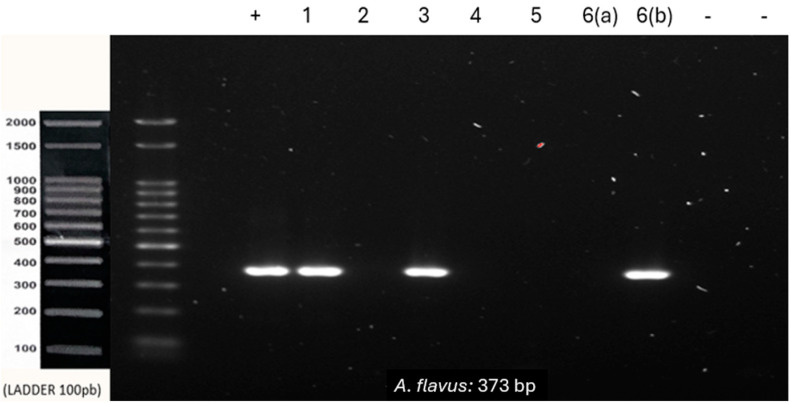
PCR using *A. flavus* primer with 100 ng of DNA from each sample. (+) DNA extracted from *A. flavus* culture (positive control). (-) Negative control of the reaction with and without human DNA. 1–6: Paraffin-embedded samples of lesions suggestive of aspergillosis. 6(a) and 6(b): Lesions removed from the same patient and embedded in two separate blocks.

Co-infection with *A. fumigatus* and *A. flavus* was observed in sample 1. Samples 3 and 6 were positive only for *A. flavus.* Sample 4 was positive only for *A*. *fumigatus*, whereas samples 2 and 5 were negative for all tested species (Figure 2 and Figure 3). 

**Figure 4:**
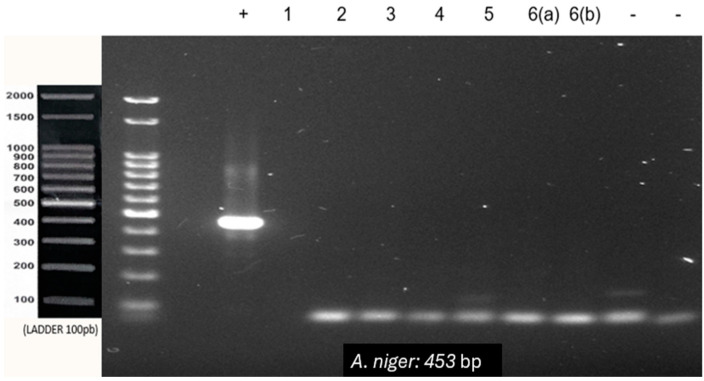
PCR using *Aspergillus niger* primer with 100 ng of DNA from each sample. (+) DNA extracted from *A. niger* culture (positive control). (-) Negative control of the reaction with and without human DNA. 1–6: Paraffin-embedded samples of lesions suggestive of aspergillosis. 6(a) and 6(b): Lesions removed from the same patient and embedded in two separate blocks.

All the samples tested negative for *A. niger* (Figure 4). The inflammatory fibrous hyperplasia samples were negative for all three species tested, reinforcing the specificity of the primers and lack of contamination during the reaction setup. The results are summarised in Table 2. 

**Figure 5:**
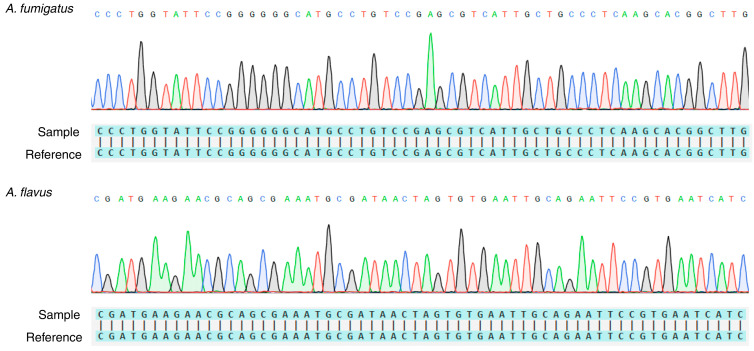
Sanger sequencing results and sequence alignment to reference genomes.

Regarding the Sanger sequencing, generated sequences displayed 100% percent identity with the respective species that were found in the investigated samples. Representative chromatograms and sequence alignments are shown in Figure 5.

## 4. Discussion

PCR has become increasingly popular as a potential diagnostic tool for several infectious diseases, since it is faster than traditional methods and provides good sensitivity and specificity [22]. However, the early diagnosis of aspergillosis remains highly challenging. Its signs and symptoms are nonspecific, and the gold standard test, which is culture, can take a long time to provide definitive and assertive results. The second most commonly used diagnostic tool in these cases is histopathological analysis, which has significant limitations in terms of specificity and sensitivity, because the arrangement of hyphae may resemble other fungal infections, thus requiring deep knowledge from pathologists to establish a differential diagnosis [19,23]. 

With the advancement of SARS-CoV-2 and the possibility of co-infection with aspergillosis, the mortality and morbidity rates associated with fungal infections have increased since 2019 [8,20]. This scenario warrants the need for obtaining a rapid and accurate diagnosis capable of optimising and adequately directing the therapeutic management of these patients to minimise the risk of infection. 

Aspergillosis is also of dental interest, since filamentous fungi, such as *Aspergillus*, have been isolated in root canals of teeth with pulp necrosis and apical periodontitis. Such endodontic conditions in the upper posterior teeth are strongly associated with maxillary sinusitis [15].

Despite recent advances in molecular studies as a diagnostic tool for many diseases, its application in aspergillosis is still incipient and restricted [22]. Its major limitation is the lack of standardization to ensure reproducibility, specificity, and sensitivity [10,21].

Aspergillosis is also of dental interest, since filamentous fungi, such as *Aspergillus*, have been isolated in root canals of teeth with pulp necrosis and apical periodontitis. Such endodontic conditions in upper posterior teeth are strongly associated with maxillary sinusitis [15].

Despite advances in molecular studies in recent times as a diagnostic tool for many diseases, its application to aspergillosis is still incipient and restricted [22]. Its major limitation is the lack of standardization to ensure reproducibility, specificity, and sensitivity [10,21].

We made the following main changes made in the sample processing protocol and in the traditional sequence of gDNA extraction kits commonly used in molecular biology: freezing and agitation with 3 mm diameter beads to promote disruption of the fungal cell wall composed mainly of chitin; increasing the concentrations of the reagents used in the protocol, for example, 40 μL of proteinase K instead of 20 μL as suggested by the manufacturer. Therefore, we sought to combine chemical and mechanical methods to obtain the highest possible concentration of gDNA from the samples.

Of the more than 200 existing species of *Aspergillus*, we selected the three most important in the human pathological context [24]. We searched for specific ITS regions in each species, resulting in three primers that allowed us to identify *Aspergillus* in the selected samples. Testing these primers in positive and negative control samples ensured their specificity for the sequences of interest.

This study also allowed us to observe the possibility of co-infection with different species in the same lesion, which may have a significant clinical impact, given that distinct species may exhibit variable susceptibility to antifungal drugs. For example, *A. flavus* is more resistant to antifungal drugs than other known species [24]. Although our study showed that PCR can be an auxiliary tool for diagnosing aspergillosis, the low number of samples and the fact that none of the cases had the diagnosis confirmed by culture are limiting factors that should be considered. Finally, information on patient management and treatment was not available because of the retrospective nature of this study. Therefore, further prospective investigations using a larger significant number of samples are needed to validate the results presented here and to clarify the role of PCR in the clinical management of the disease. In addition, a comparative analysis of PCR with other diagnostic tests for aspergillosis is essential to evaluate the accuracy of the standardised protocol.

## 5. Conclusions

In conclusion, we showed in the present study that the clinical/pathological diagnosis of aspergillosis can be enriched by PCR, especially in challenging cases or cases where the patient is resistant to first-choice treatment. We also showed that co-infection with different *Aspergillus* species can occur during the oral manifestation of the disease. Further studies with a larger sample size, in which cell culture could be employed as a comparative diagnostic method, are vital for analysing the accuracy of PCR in diagnosing aspergillosis. 

## Figures and Tables

**Table 1:** Sequence of primer pairs for each species studied.

Species	Primer Sequence (5′-3′)	Amplicon Size
*A. fumigatus*	F-GTCTGAGTTGATTATCGT	312 bp
	R-GGCCTACAGAGCAGGTGAC	
*A. flavus*	F-CACCACGAACTCTGTCTGATC	373 bp
	R-GATTGATTTGCGTTCGGC	
*A. niger*	F-GCCCAACCTCCCATCCGTG	453 bp
	R-CAATCCTACAGAGCATGTG	

F: forward primer R: reverse primer.

**Table 2:** PCR results of each sample by species.

Samples	Location	Sex	Age	PCR Test Results		
				*A. niger*	*A. fumigatus*	*A. flavus*
1	Maxillary sinus	M	79	-	+	+
2	Maxillary sinus	M	55	-	-	-
3	Maxillary sinus	M	49	-	-	+
4	Upper gum	M	56	-	+	-
5	Frontal sinus	M	57	-	-	-
6	Maxillary sinus	M	52	-	-	+

M = male; Age = years old.

## Data Availability

The raw data supporting the conclusions of this article will be made available by the authors on request.
